# Hippocampal Neurogenesis and Antidepressive Therapy: Shocking Relations

**DOI:** 10.1155/2014/723915

**Published:** 2014-05-22

**Authors:** Peter Rotheneichner, Simona Lange, Anna O'Sullivan, Julia Marschallinger, Pia Zaunmair, Christian Geretsegger, Ludwig Aigner, Sebastien Couillard-Despres

**Affiliations:** ^1^Paracelsus Medical University, Institute of Experimental Neuroregeneration, Spinal Cord Injury and Tissue Regeneration Center Salzburg (SCI-TReCS), Strubergasse 22, 5020 Salzburg, Austria; ^2^Paracelsus Medical University, Institute of Molecular Regenerative Medicine, Spinal Cord Injury and Tissue Regeneration Center Salzburg (SCI-TReCS), Strubergasse 22, 5020 Salzburg, Austria; ^3^University Clinics of Psychiatry and Psychotherapy I, Paracelsus Medical University, Ignaz-Harrer-Straße 79, 5020 Salzburg, Austria

## Abstract

Speculations on the involvement of hippocampal neurogenesis, a form of neuronal plasticity, in the aetiology of depression and the mode of action of antidepressive therapies, started to arise more than a decade ago. But still, conclusive evidence that adult neurogenesis contributes to antidepressive effects of pharmacological and physical therapies has not been generated yet. This review revisits recent findings on the close relation between the mode(s) of action of electroconvulsive therapy (ECT), a powerful intervention used as second-line treatment of major depression disorders, and the neurogenic response to ECT. Following application of electroconvulsive shocks, intricate interactions between neurogenesis, angiogenesis, and microglia activation, the hypothalamic-pituitary-adrenal axis and the secretion of neurotrophic factors have been documented. Furthermore, considering the fact that neurogenesis strongly diminishes along aging, we investigated the response to electroconvulsive shocks in young as well as in aged cohorts of mice.

## 1. Electroconvulsive Therapy and Major Depression Disorders

Major depression is one of the most common forms of mental disorders in humans. Hereditary and environment-triggered forms of this disease lead to a severe decline of life quality and might elicit life-threating events like suicide attempts. Despite the constant development and improvement of antidepressive drugs, a compound providing fast and long-lasting pharmacological efficacy in most patients is still lacking. Although the binding specificity for recently developed drug-classes, for example, selective serotonin noradrenalin reuptake inhibitors (SSNRIs), has been improved further, concerns regarding their side effects, such as the increased risk of suicide, must be heeded.

Electroconvulsive therapy (ECT) as an alternative treatment offers a safe, rapid, and potent therapy to severely depressed patients. ECT-associated side effects, like cognitive impairments, are transient and may slightly vary from one ECT-protocol to the other [1, 2]. ECT was firstly performed by Ugo Cerletti and Lucio Bini in 1938 [3] and induces rapid improvements on mental disorders. In contrast, standard pharmacological antidepressive treatments, such as the administration of selective serotonin reuptake inhibitors (SSRIs), commonly show their first efficacy weeks after initiation and are dependent on individual features such as sex, treatment, or disease pattern [4, 5]. Since the introduction of ECT in the clinic, several parameters, such as electrode placement, pulse width, and pulse shape were improved in order to optimize efficacy and minimize side effects [6–8]. Nowadays, ECT has several indications, for instance, psychotic and melancholic depressions, malignant catatonia, and delirious mania [9]. Nevertheless, ECT is predominantly used in severe cases of major depression as a second line treatment following failure of pharmacological approaches [10]. Overall, according to meta-analyses of clinical studies ECT was reported to be significantly more effective in depressive disorders compared to pharmacological treatments [2, 11].

## 2. Are Neurogenesis and Antidepressive Activity Linked?

Adult neurogenesis, the generation of new neurons, takes place in numerous mammalian species, including humans [12, 13]. Neurogenesis occurs in at least two specific areas of the adult brain, namely, the dentate gyrus of the hippocampus and the subventricular zone (SVZ) of the lateral ventricles. In these so-called neurogenic niches, neural stem cells (NSCs) can on the one hand divide symmetrically, leading to two daughter stem cells and on the other hand perform an asymmetric division resulting in a daughter stem cell and a rapid amplifying progenitor cell. Depending on the microenvironment and surrounding factors, these progenitor cells can, over a period of three to four weeks in rodents, mature into neurons which integrate into existing neuronal networks. Along aging, the number of neural stem and progenitor cells declines significant and various steps of neurogenesis (e.g., proliferation, survival, or maturation) get decelerated [14–16]. The cause for the decreased levels of proliferation in old individuals is still under debate. Putative mechanisms could lengthen cell cycles, deplete progenitor cells in the hippocampus, or cause their quiescence [14]. Additionally, events such as depression or stress strongly reduce the hippocampal potential for plasticity [17]. Environmental changes, however, can counteract this decline by stimulating neurogenesis and probably lead to healthy aging [18, 19].

To elucidate the mechanisms by which ECT leads to antidepressive effects, preclinical research has primarily made use of experimental electroconvulsive shock (ECS), the animal model counterpart of ECT. A strong enhancement of neurogenesis has been observed in various species following experimental ECS treatments [20, 21]. Several studies indicated a close relation between hippocampal function and mood regulation. The observation of an antidepressive-like effect and an upregulation of hippocampal cell proliferation upon experimental ECS raised speculations on the participation of neurogenesis in the antidepressive mode of action. However, evidence for a direct participation of neurogenesis in antidepressive mechanisms still remains to be convincingly demonstrated [17].

Based on various correlations observed in experimental ECS animal studies, several hypotheses like the stimulation of neurogenesis, the restoration of hippocampal volume, modulations of neurotransmitter and hormone levels, changes in angiogenesis, and cerebral blood flow have been formulated to account for the antidepressive action of ECT. In this report, we revisited the impact of experimental ECS on neurogenesis and speculate on the mechanisms leading to its upregulation, which may be also involved in ECT's antidepressive effects in patients. Moreover, the influence of aging on the impact of ECT/experimental ECS and neurogenesis has been scrutinized in more detail.

## 3. Enhanced Neurogenesis following Antidepressive Treatment: Effect or Side Effect of the Therapy?

Numerous groups reported a close relation between adult born hippocampal neurons and antidepressive effects [22]. Chronic administration of antidepressants, which counteract the negative behavioural effects of chronic stress, leads reportedly to an upregulation of neurogenesis [23]. Additionally evidence supporting the involvement of neurogenesis in the mode of antidepressant action has subsequently been revealed by Santarelli and colleagues, showing that deletion of neurogenesis in mice via hippocampal X-ray irradiation ablated the behavioural response to the antidepressants imipramine and fluoxetine [24]. However, further experiments displayed that the ablation of hippocampal neurogenesis* per se* does not elicit a depressive phenotype [25–28]. Besides, it could be shown by Sah and colleagues, using a mouse model of increased anxiety, that rather the levels of neuronal activity in the dentate gyrus than the rates of neurogenesis correlate with the depression-like behaviours [29].

To evaluate studies addressing experimental ECS working mechanisms, it is noteworthy that most studies have been performed in healthy animals and only occasionally in animal models based on chronic stress. These situations certainly differ from the complex conditions pertaining in patients with mental disorders like major depression. Despite this, biological effects of experimental ECS on the central nervous system (CNS) in animals can be monitored properly. Taken together, the accumulated evidence suggests that depression does not result from an impaired neurogenesis but that the addition of new neurons in the hippocampal circuitry may facilitate the action of antidepressants.

It is intriguing that the delayed behavioural response to common antidepressant drugs, for example, SSRIs, in rodents coincides with the delayed upregulation of neurogenesis, that is, after approximately three weeks [30]. Similarly, the rapid antidepressive action of experimental ECS is associated with a rapid stimulation of neurogenesis, which follows after the first treatment [31]. In this context, the enlargement of the immature neuron population may be involved in the behavioural response. These immature cells have been demonstrated to be highly excitable compared to mature neurons [32]. Therefore, stimulation by lower input intensity could lead to a higher input sensitivity of the whole surrounding network.

Nonetheless, one should not get the impression out of these speculations that depression is exclusively a hippocampal disorder. The involvement of additional cerebral structures, such as the amygdala, the frontal cortex, and the thalamus, has been well documented in studies revealing structural abnormalities and disbalances of neurotransmitters [33–35]. Hence, ECT is likely to act on numerous cerebral structures, possibly stimulating their neuronal plasticity. In this respect, pharmacological antidepressive treatments were shown to restore the mechanism of long term potentiation (LTP) [36, 37], which is impaired in depressed patients and animal models with depressive-like behaviours. As previously demonstrated for pharmacological treatments, ECT might stop dendritic atrophy [38, 39] or increase dendritic spine density in various cortical and limbic structures [40, 41]. In the hippocampus, plasticity is not restricted to synaptic plasticity but also involves cellular plasticity, that is, neurogenesis. As mentioned above, an enhanced number of immature neurons may increase excitability of the hippocampus and putatively associated areas of the limbic system as well. Alternatively or in addition, ECT might rewire existing neuronal networks, for example, via sprouting of mossy fibers, that is, axonal projections of the granular neurons to the CA3 pyramidal neurons in the hippocampus, restoring thereby functionality of limbic and cortical circuits. Although it remains to be definitely demonstrated that upregulation of neurogenesis contributes to the antidepressive action, levels of neurogenesis could become a surrogate marker to predict and monitor the patients' response to treatment. Even though adequate protocols for* in vivo* measurement of human neurogenesis are not existent yet, efforts have been recently deployed using magnetic resonance spectroscopy (MRS) [42, 43] and positron emission tomography (PET) using labelled tracer [44].

## 4. Differences in ECS and Pharmacological Treatment on Neural Stem Cells

**Figure 1:**
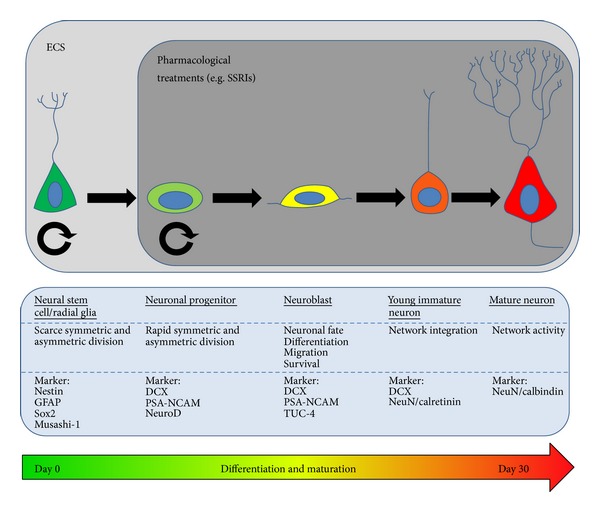
Differentiation and maturation of adult neural stem cells. The process from stem cell division to the fully integrated mature neuron in the dentate gyrus takes approximately 30 days in rodents. Specific markers characterize the various maturation stages (e.g., DCX for neuroblasts). Experimental ECS or pharmacological treatments can influence neurogenesis at various stages; experimental ECS is also reported to activate quiescent stem cells.

The finding that experimental ECS strongly induces hippocampal neurogenesis in various mammals, including rodents and nonhuman primates, triggered the idea of using ECS to modulate the pool of neural stem and progenitor cells. Pharmacological antidepressive treatments, like fluoxetine administration, act mainly on amplifying neural progenitors (ANPs) [45]. In contrast, it has been postulated that experimental ECS can in addition shove quiescent neural progenitors (QNPs) from their resting phase to a proliferative status, thereby increasing the pool of active stem cells generating new progenitor cells [46, 47] (Figure 1). Importantly, new neurons generated upon experimental ECS-treatment do not differ in fate or phenotype as compared to neurons generated under physiological conditions [31]. Hence, new neurons generated following experimental ECS mature and integrate in the existing neuronal network and form appropriate synapses [48, 49].

## 5. Age-Dependent Response to ECT

To date, most experimental ECS studies were performed in young animals, even though depressive episodes must be addressed also in aged individuals. As a group, in which major depression disorders are increased and often underdiagnosed, the elderly could especially benefit from ECT. On the one hand, this population is often poly-medicated, which increases the risk of adverse drug interaction upon additional pharmacological interventions. On the other hand, ECT offers an alternative to classical pharmacological antidepressants, which fail as first line treatment in the aged population in up to 77% [50]. This lack of response to antidepressant drugs might be exacerbated by age-related physiological changes, such as decreased concentrations of acetylcholine and dopamine, increased level of monoamine oxidase activity, or increased concentration of cortisol due to a dysregulation of the hypothalamic-pituitary-adrenal (HPA) axis [50]. Remarkably, repeated ECT treatments have shown to be as effective as pharmacological antidepressive therapy in avoiding relapse in aged individuals [51]. Nevertheless, both pharmacological and ECT-based approaches benefit from a multimodal therapy concept complemented by physical and mental exercises, psychological therapy, and resocialisation.

**Figure 2:**
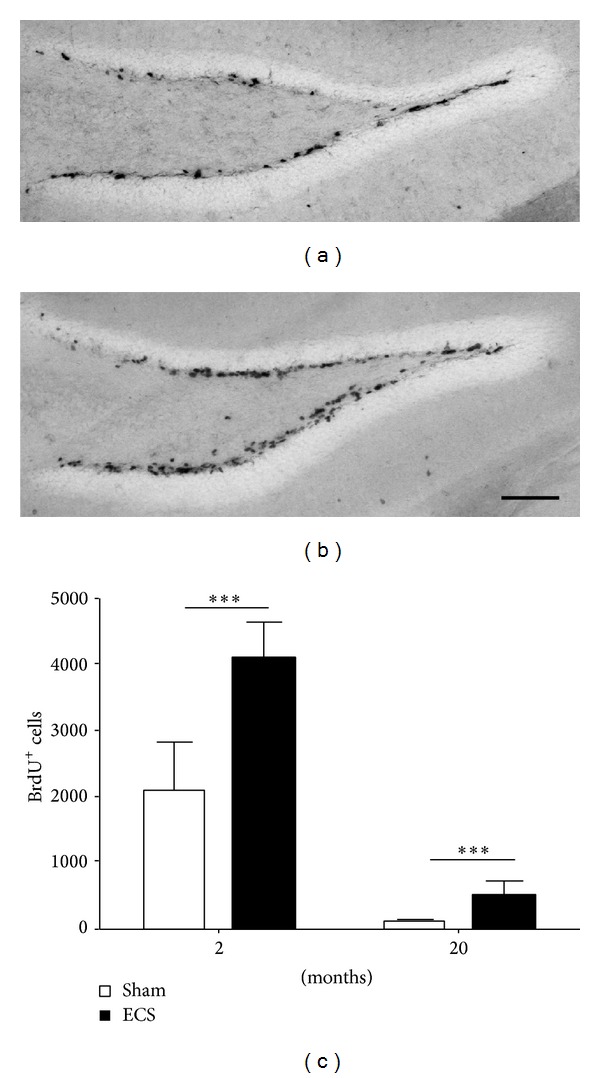
Representative micrographs showing the detection of BrdU-labelled cells in the dentate gyrus of a 2-month-old mouse from (a) the sham group or (b) the experimental ECS group. Scale bar: 200 *μ*m. (c) The number of BrdU^+^ cells was determined to estimate the rates of cell proliferation in the dorsal DG of 2- and 20-month-old mice. Both groups showed a highly significant enhancement in cell proliferation following experimental ECS-treatment. Two months, *P* < 0.0001 (***); 20 months, *P* = 0.0004 (***).

So far, the mechanisms by which experimental ECS and antidepressant drugs interfere with cells of the neurogenic niches could not be fully deciphered. Both types of antidepressive treatments have been reported to increase the number of proliferating cells in the hippocampus and promote the maturation of neuronal progenitors [5, 20, 30, 52]. Under our experimental ECS paradigm, we could demonstrate a highly significant increase of cell proliferation in the dentate gyrus of mice from the young and aged groups (for details please see section Material and Methods) (Figure 2). In the 20-month-old mice, ECS increased the number of BrdU-labelled cells by more than sixfold as compared to the aged sham-treated mice, whereas in the 2-month-old mice the increase was roughly a doubling. Most of the BrdU^+^ cells were located in the subgranular zone of the dentate gyrus, the place where neural stem cells are resident. Intriguingly, those BrdU^+^ cells in the 20-month-old group expressed neither Nestin, a marker for neural stem cells, nor Doublecortin (DCX), a marker for neuronal progenitors. It is possible that in aged individuals the maturation steps from stem cells and early progenitors to immature neurons expressing DCX are slowed down. Further studies need to address kinetics and fate of newly generated cells after experimental ECS, especially in aged individuals.

## 6. How Neurogenesis May Be Supported by Modulation of Microenvironment

**Figure 3:**
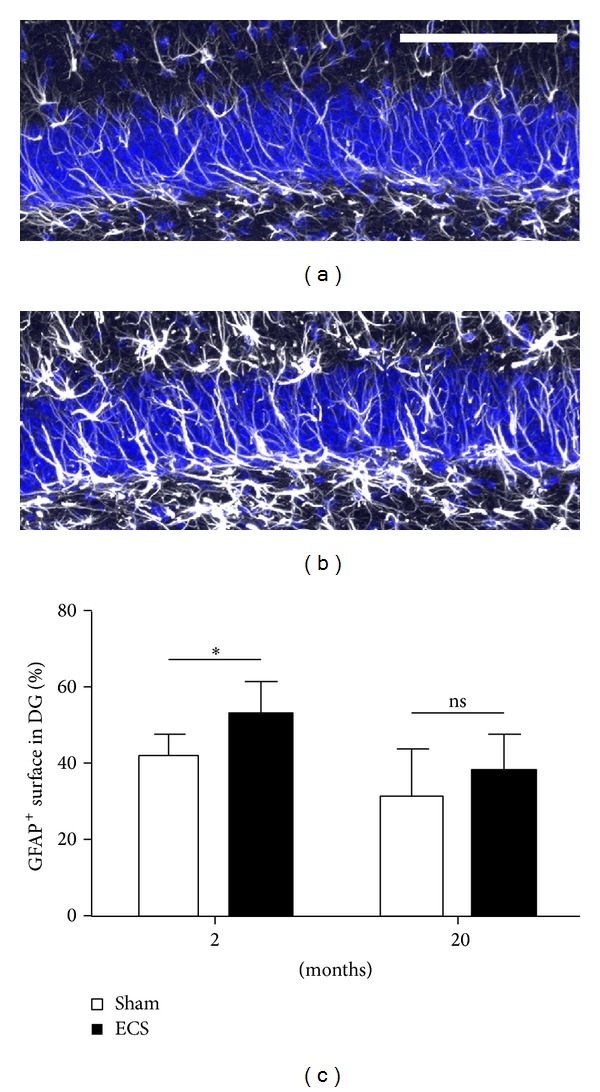
Detection of GFAP in the dentate gyrus of (a) sham-treated or (b) ECS-treated mouse of the 2 months of age cohort, scale bar in (a) 100 *μ*m. (c) Graph showing percentage of area covered by GFAP-expressing cells in the granular layer of dentate gyrus in 2- and 20-month-old mice following ECS or sham treatment. A two-way ANOVA detected a significant increase of the GFAP expression upon experimental ECS (*P* = 0.0043 (**)). Bonferroni post hoc test revealed that the significance resulted from differences in the 2-month-old group (*P* < 0.05).

Neural stem and progenitor cells are embedded in a complex cellular niche with tight regulatory interactions between the different cellular partners. Not only is this microenvironment exposed to factors delivered by the blood system but also stem and progenitor cells are in close contact with cells forming and surrounding the blood vessels such as pericytes and astrocytes [15, 53]. This organization referred to as the neurovascular unit is involved in the precise regulation of neurogenesis [54]. It is well documented that endothelial cells, astrocytes, and microglia modulate the behaviour of stem and progenitor cells [55–57]. The impact of experimental ECS on these cell populations was therefore examined. In mice and nonhuman primates, histological staining revealed that experimental ECS increases the expression level of GFAP as well as the number of cells expressing this astrocyte marker in the hippocampus [20, 47, 58]. In our own experiments, we focused on GFAP^+^ cells in the granular layer of the dentate gyrus of 2- and 20-month-old mice. Overall, experimental ECS-treatment increases the expression of GFAP significantly (Figure 3). A detailed analysis of our data showed an age dependent effect of this treatment; hence, only the 2-month-old group of mice showed a significant increase of GFAP-expression. Some recent studies demonstrated that GFAP-expressing cells in the cortex could be triggered to become multipotent neural progenitors [59]. It remains to be elucidated if experimental ECS and ECT can recruit a pool of quiescent progenitors within the hippocampus and thereby increase neurogenesis.

**Figure 4:**
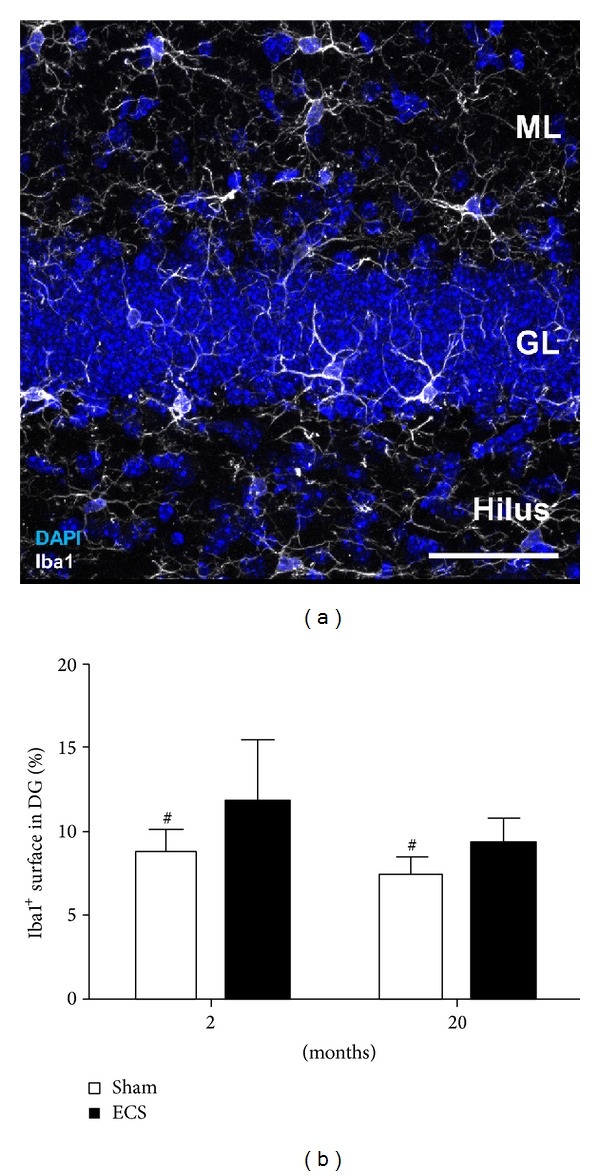
(a) Detection of microglia in the granular layer (GL) of a 2-month-old mouse based on the expression of Iba1 (white) (ML: molecular layer). Scale bar: 50 *μ*m. (b) Microglia volume was estimated according to the percentage of the surface covered by Iba1^+^ labelling in the granular layer of 2- and 20-month-old mice following ECS or sham treatment. Two-way ANOVA revealed upon ECS a significant increase of Iba1-labeled surface in the dentate gyrus of both age groups (*P* = 0.0475).

Microglia, the immune cells of the CNS, have been shown to influence neurogenesis [60]. Within the granular layer in the dentate gyrus of young and old mice we detected cells, expressing the ionized calcium-binding adaptor molecule 1 (Iba1), a marker for microglia cells. With our experimental setup, we observed no increase in the amount of Iba1^+^ cells in the dentate gyrus (2 months: sham 13885 ± 1606 cells/mm^3^ versus ECS 12656 ± 2480 cells/mm^3^ *P* = 0.511; 20 months: sham 10753 ± 955 cells/mm^3^ versus ECS 11750 ± 1696 cells/mm^3^ *P* = 0.385); however, the experimental ECS enhanced the volume of Iba1^+^ cells significantly (Figure 4). These enlarged microglia, namely, hyper-ramified, constitute an intermediate state between resting and activated [61]. Such enlarged microglia have been previously reported to appear after experimental ECS-treatment in rodents [62]. Furthermore, Jansson et al. reported an upregulation of MHCII in microglia following experimental ECS, which also suggests an activation of these immune cells. The group proposed that these MHCII-expressing microglia play a significant role for maintenance of neurogenesis in the hippocampus. Taken together, these observations indicate that experimental ECS modulates the microglial activation state to the benefit of neurogenesis and therefore potentially contributes also to the antidepressive effect.

## 7. Blood System and Its Role in ECT

The antidepressive action of ECT can most likely not be reduced to a single mechanism, but results from the synergistic cooperation of several processes and factors involving neurogenesis, modulation of blood supply and angiogenesis, corticosteroids, brain-derived neurotrophic factor (BDNF), vascular endothelial growth factor (VEGF), and so forth. Indeed, a closer look at these putative modes of action reveals close interconnection between the different systems. For instance, proliferation of stem cells located in the neurogenic niches can be modulated by factors delivered via the closely associated vasculature [63]. In aged mice, we showed that neurogenesis is downregulated by blood derived signalling molecules that are delivered through the vessels. However, neurogenesis can be reactivated by the introduction of young blood into the circulation via direct serum injection or heterochronic parabiosis [64].

Considering that neurogenic niches are closely apposed on blood vessels delivering nutrients and numerous factors, it is noteworthy that comorbidities between cardiovascular diseases and depression have been reported in numerous studies (e.g., see [65, 66]). Moreover, PET imaging revealed that ECT treatment transiently changes local cerebral blood flow, leading to an increase in the brain stem, diencephalon and basal ganglia for instance [67]. Yet, the so-called blood-brain barrier (BBB) constitutes a barrier controlling the molecular exchange between the circulating blood and the brain parenchyma (reviewed, e.g., by Ilbay and colleagues [68]). Roughly speaking, the BBB prevents under physiological conditions the diffusion of large and/or hydrophilic molecules but allows for the passage of hydrophobic molecules and substances having their specific transporters, for example, glucose. Neuropathological disturbances like stroke or epileptic seizure, can lead to leakage of this barrier [68]. Under these conditions, various circulating molecules can diffuse out of the blood vessels and thereby change the microenvironment of the brain parenchyma and of the neurogenic niches.

It has been proposed that experimental ECS leads to a greater permeability of the BBB, enabling an influx of blood-derived factors into the parenchyma. However, leakage of the BBB has not been observed as an acute effect of experimental ECS but was rather seen following several experimental ECS sessions in rodent models [69]. For instance, no increased permeability to Evans Blue was reported by Oztas and collaborators following a single experimental ECS treatment; however, BBB leakage could be recognized after ten experimental ECS-sessions [70]. The potential BBB leakage after ECT was furthermore analysed in humans using different tracers for PET imaging by Bolwig and colleagues, who concluded that, due to changes in local cerebral blood flow and potentially from the leakage of newly formed capillaries, proteins could pass more easily out of the blood vessels following ECT [71]. In a subsequent study on the experimental ECS-induced BBB leakage in rats, Sartorius and colleagues reported elevated BDNF levels in the serum as well as the prefrontal cortex and the hippocampus after five experimental ECS sessions. According to their hypothesis, a fraction of the parenchymal BDNF originated from the blood circulation and crossed the BBB after experimental ECS [72]. Hence, enhanced BBB permeability might contribute to the beneficial impact of repeated ECT session, yet the rapid induction of neurogenesis seen after a single experimental ECS session is unlikely linked to an early failure of the BBB integrity.

**Figure 5:**
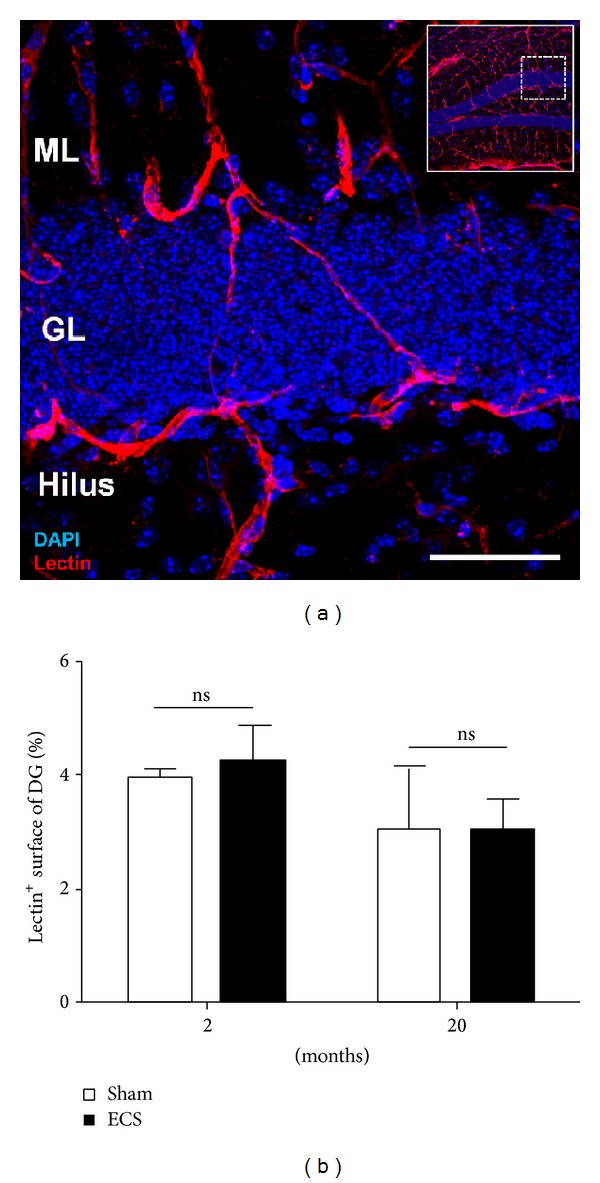
(a) Detection of blood vessels in the granular layer of the dorsal dentate gyrus with B. simplicifolia lectin (red) (inset, overview of the dentate gyrus with position of the field of view, ML: molecular layer, GL: granular layer). Scale bar: 50 *μ*m. (b) No significant differences in blood vessel density could be detected following ECS. Interestingly, aged brains had a slightly decreased vessel density compared to the younger group (*P* = 0.0122).

Strikingly, Hattiangady and colleagues reported that the number of neural stem cells in the dentate gyrus is not decreasing with age, but only their activation state, which was shown to be closely dependant on the distance of stem cells to the next blood vessel [73]. Therefore, experimental ECS might upregulate neurogenesis by promoting a closer association of stem cells to the blood vessels. Angiogenesis, the generation of new blood vessels, is tightly regulated during development and in the adult brain by numerous factors in a similar fashion to neurogenesis [74]. In the healthy brain, the density of blood vessels remains relatively stable during adulthood [75]. However, increased angiogenesis (and neurogenesis) was observed in SSRI-treated patients suffering from major depression [74]. Similarly, experimental ECS treatment was reported to promote the elongation of existing vessels and to increase their density in the stratum lacunosum moleculare (SLM), a subregion of the hippocampal molecular layer [76, 77]. However, within the granular layer of the dentate gyrus, that is, in the vicinity of stem and progenitor cell somas, we did not detect an increase of the blood vessel density upon experimental ECS treatments (Figure 5). Nevertheless, an enhanced vascularization in the molecular layer could intensify the delivery factors to the tip of radial stem cells or to the dendritic trees of granular neurons for retrograde transport and signalling. Alternatively, factors secreted in the molecular layer could act on other cells types capable of neurogenesis modulation, for example, astrocytes and microglia. Furthermore, Newton et al. proposed that endothelial cells* per se* can influence neurogenesis by secreting factors acting on neural stem and progenitor cells [77].

## 8. Neurogenesis and the Normalisation of the HPA-Axis

The crosstalk between neurogenesis and the ECT putative modes of action is further exemplified by the modulation of the hypothalamic-pituitary-adrenal-axis (HPA-axis) signalling during depression and its interaction with the neurogenesis [78–81]. In depressed patients, the regulation of the HPA-axis is heavily disturbed. The malfunction of the negative feedback mechanisms results in an excessive secretion of the hormone cortisol. Interestingly, increased levels of cortisol and defective HPA-axis response were also reported in aged individuals [82]. In general, the level of glucocorticoids negatively correlates with the proliferation rates of neural stem and progenitor cells which possess the receptors for glucocorticoid and mineralocorticoids [83, 84]. Surget and colleagues established an important link between neurogenesis and the antidepressive effect of fluoxetine by identifying a direct correlation between newly generated neurons and the restoration of adequate control on the HPA stress response system [85]. The report demonstrated namely that the generation of new neurons is required for the function of the negative feedback loop of the HPA-axis, while others critically discuss a direct correlation [86]. Interestingly, high levels of cortisol have also antiangiogenic properties [87]. Long-lasting elevated cortisol levels, such as in the aged individual, have been proposed to induce epigenetic alterations in neural stem and progenitor cells, which could lead to their long term quiescence and therefore to a significant reduction of neurogenesis [88].

Preclinical research on experimental ECS has been performed often in young and healthy animals, which do not show signs of depressive behaviour. For a better understanding of the processes taking place in the depressed brain and the impact of ECT on the CNS, adequate animal models showing depressive-like behaviour with elevated corticosteroid-levels should be selected. This could be achieved artificially by direct injection of corticosteroids. Application of large doses of corticosteroids was reported to inhibit neurogenesis in a rat model. In this model, cell proliferation in the dentate gyrus could be restored to physiological levels by experimental ECS treatment [89]. Hence, experimental ECS may be indirectly involved in the normalization of the HPA-axis regulation through the activation of neurogenesis.

## 9. Release of Signalling Factors following Experimental ECS and ECT

In animal models, experimental ECS activates additionally neurons of the hypothalamic-pituitary-system, namely, the paraventricular nucleus, supraoptic nucleus, and ventromedial nucleus (PVN, SON, and VMH), which regulate the secretion of diverse depression-relevant HPA-axis-related hormones, such as adrenocorticotropin, neuropeptide Y (NPY), prolactin, and vasopressin [62, 90–94]. It has been reported that NPY concentrations were decreased in the cerebrospinal fluid (CSF) of depressed patients, but through ECT treatments the level could be normalized again [95–97]. The link between NPY and depression was further validated in rodents, in which a direct administration of NPY into the lateral cerebral ventricles led to an antidepressive effect [94].

In addition to the modulation of HPA-related neuropeptides, experimental ECS treatment leads to higher concentration of several neurogenic/neurotrophic factors like BDNF, VEGF, neuritin, nerve growth factor (NGF), and fibroblast growth factor 2 (FGF2) within the CNS [46, 98–100]. A stronger secretion of these factors and the induction of cell proliferation can be observed even after a single experimental ECS; however, the proliferation level will further increase with multiple experimental ECS sessions [21, 101, 102]. The biological activities of factors induced by experimental ECS are manifold. For instance the neurotrophic factor neuritin, which is one of the most upregulated genes in the granule cell layer of the dentate gyrus following experimental ECS, induces neurite outgrowth [103]. Other upregulated factors, like FGF-2, possess mitogenic, neurotrophic, and neuroprotective capabilities [104]. Finally, VEGF, besides being one of the most potent angiogenic factors, also promotes neurogenesis after experimental ECS [46, 63, 105, 106].

Importantly, experimental ECS treatments increase the levels of BDNF in areas of the brain relevant to depression, such as the entorhinal cortex and the hippocampus [99, 107]. The observation that the infusion of BDNF into the midbrain or hippocampus led to an antidepressive effect substantiated the role of BDNF in experimental ECS mode of action [108, 109]. BDNF was also shown to increase synaptic strength, neuronal survival, and integration but not the proliferation [110, 111]. In animal models receiving chronic administration of antidepressant drugs, the appearance of the antidepressive activity coincided with the increase of BDNF concentrations and relied on TrkB-signalling [112]. Interestingly, secretion of proBDNF, the precursor of mature BDNF, was increased in rat hippocampal synaptosomes even after one experimental ECS session and could be further enhanced by repeated sessions [113].

The neurogenic impact of BDNF not only is dependent on its net concentration in the brain but also is determined by the BDNF subtypes expressed. BDNF has at least 34 different transcripts, which can be translated to mature forms of BDNF [114]. Alternative and age dependent regulation in various brain regions and involvement of BDNF in diverse functions complicated the analysis of BDNF signalling [115]. It is noteworthy that a polymorphic variant of BDNF (G169A) might associate with major depression and carriers of this polymorphism were observed to have a reduced hippocampal volume. Nevertheless, these individuals do not differ in their response to ECT in comparison to other depressed patients [116].

## 10. Gain of Hippocampal Volume following Experimental ECS and ECT

Several studies pointed out a significant hippocampal atrophy in depressed patients and animal models of depression [117–121]. This decline of hippocampal volume could be directly correlated with the duration of untreated depression [120]. Postmortem studies revealed that the hippocampal volume reduction originated from a reduction of neuronal volume and of the glial density [122–124]. Nevertheless, a normal hippocampal volume could be regained following ECT-treatment of depressed patients [125]. Interestingly, the hippocampal volume could also be significantly increased by ECS session in nondepressed rodent and non-human primate models [20].

**Figure 6:**
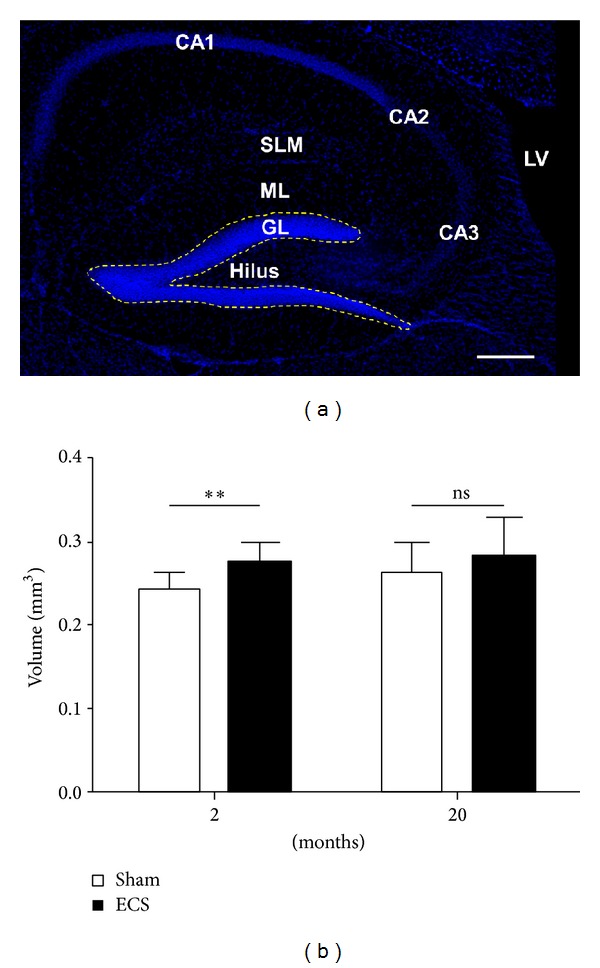
(a) Overview of a murine hippocampus with its substructures* cornu ammonis* (CA) 1–3, lateral ventricle (LV),* stratum lacunosum moleculare* (SLM), molecular layer (ML), granular layer (GL), and the hilus. Scale bar: 200 *μ*m. (b) Volume of the dorsal dentate gyrus of 2- and 20-month-old mice following ECS or sham-treatment. Young mice showed a significant increase of volume after ECS (2 months, *P* = 0.002 (**); 20 months (ns)).

As mentioned above, the brains of young and old differ in several aspects, including changes in neurotransmitter concentrations, decline of stem and progenitor cell proliferation, or alterations of hormone levels. In our experiments, experimental ECS treatments could slightly, but significantly, increase the volume of the dentate gyrus of the younger cohort of mice (Figure 6). In the 20-month-old mice the volume increase failed to reach significance. Hence, the mechanisms leading to the volumetric changes following experimental ECS appear to be dampened with ageing, which may disclose a reduced plasticity and correlate with the higher rates of depression recurrence observed in the elderly population [126]. Although the validity of volumetric measurements as a biomarker for the monitoring of depression remains to be established, the rapid volumetric response to experimental ECS treatment may reflect the responsiveness of mechanisms controlling plasticity of the brain.

## 11. Epilepsy and Experimental ECS/ECT-Induced Plasticity

ECT shares some similarities with epileptic seizures, for instance, a massive excitation of neuronal networks and an enhanced cell proliferation in dentate gyrus. It is noteworthy that experimental ECS, in contrast to epileptic seizures, results neither in neurotoxicity and apoptosis, nor in the appearance of ectopic neurons in the hilus of the hippocampus [21, 76]. A proper wiring between the dentate gyrus and its target area, namely, the CA3, is important for correct processing of information entering the hippocampal circuitry. This connectivity has been reported to be aberrant in the brains of patients suffering of temporal lobe epilepsy or following experimental seizures in rodents [127]. On the other hand, experimental ECS sessions promote sprouting of mossy fibers without generation of abnormal connectivity and therefore do not lead to the appearance of spontaneous epileptic activities [128, 129]. This is in clear contrast with the stimulation of hippocampal neurogenesis through epileptic seizures, which results in the generation of new neurons characterized by structural and functional abnormalities [130].

The addition of new neurons and rewiring of hippocampal networks likely contribute to the antidepressive activity of experimental ECS, although the mechanisms are still to be elucidated. Recent evidence suggests that the antidepressive effect is not proportional to the number of newly generated neurons but rather to the activation of the whole neuronal population [86]. Substantiating this hypothesis is the observation that c-fos, a marker for cellular activity, is strongly upregulated in mature neurons of the dentate gyrus after experimental ECS [110]. Sah and colleagues further reported that the antidepressive effects of fluoxetine in a mouse model of anxiety disorder resulted from the restoration of the hypoactive dentate gyrus back to a normal activity level and not from an elevation of the neurogenesis rate [29]. Nevertheless, taken that newly generated neurons are significantly more excitable than their mature counterparts, increasing neurogenesis may also result in the increase of global dentate gyrus activity [32, 131, 132].

## 12. Outlook

There is still an important debate on the antidepressive mode of actions of ECT, but undoubtedly this treatment is a fast and powerful therapy with few side effects and, simultaneously, a very potent enhancer of hippocampal neurogenesis. Even a decade after the report that antidepressants increase the rate of hippocampal neurogenesis, relation between the latter and the antidepressive activity could not be fully deciphered. Nevertheless, evidence for a functional relevance of adult hippocampal neurogenesis in processes of neuronal plasticity and behaviours is slowly emerging.

## 13. Material and Methods

In the experiment described in this review, we involved C57Bl/6 mice of two and twenty months of age in order to reflect the various age groups receiving antidepressive treatments. Number of mice were 11 (sham) and 11 (ECS) in the 2-month-old group and 8 (sham) and 9 (ECS) in the 20-month-old group, male and female equally distributed. Experiments were performed in conformity with the Directive (2010/63/EU) of the European Parliament and of the Council and were approved by the local animal health commission. Animals were bred in the central animal facility of the Paracelsus Medical University, Salzburg, Austria. All animals were housed under standard conditions of a 12-hour light/dark cycle with food and water ad libitum.

These mice were submitted to a daily session of experimental ECS for five consecutive days. Anaesthesia right before shock application was obtained using 2.5% isoflurane in pure oxygen. ECS parameters applied were as follows: frequency 100 pulses/sec, pulse width 0.3 ms, shock duration 1 sec, and current 18 mA using an Ugo Basil 57800 ECT Unit via ear clips moisten with Ringer's Solution. Sham-treated mice were only anaesthetized daily with isoflurane. In addition, dividing cells were marked by intraperitoneally BrdU injection (50 mg/kg bodyweight) daily at the end of every ECS or sham treatment.

For histological analysis, mice were perfused intracardially on day 6 with phosphate buffered 4% paraformaldehyde pH7.4. Thereafter, brains were further post-fixed overnight in the same solution and then cryoprotected with a 30% sucrose solution for several days. Brains were cut sagittal into 40 *μ*m thick sections using a sliding microtome on dry ice (Leica, Nußloch Germany).

Immunohistological analyses were performed as previously described [133] using the following antibodies and kits: dividing cells were labelled with rat anti-BrdU (1:500, Serotec, Puchheim, Germany) and detected with a biotin-conjugated rabbit anti-rat (1:1000, Vector, Burlingame USA) followed by the VECTASTAIN ABC System and DAB Peroxidase Substrate kit (Vector, Burlingame USA). Microglia were labelled with rabbit anti-Iba1 (1:300, Wako, Neuss Germany) and detected with donkey anti-rabbit Alexa 647 (1:1000, Milipore, Billerica USA). For blood-vessel staining, biotinylated Lectin (1:500, Sigma-Aldrich, Vienna Austria) from B. simplicifolia was used followed by Streptavidin Alexa 488 (1:400, Invitrogen, Lofer Austria). Nuclei in fluorescence stainings were marked with 4′,6-diamidino-2-phenylindole (DAPI 0.5 *μ*g/mL, Sigma-Aldrich, Vienna Austria).

Extrapolation for one brain hemisphere of the total number of BrdU or Iba1-labelled cells located in the dentate gyrus was performed by detection of BrdU in every tenth section or Iba1 in 3 randomly selected fields of view. Pictures were acquired with an Olympus IX81 (Olympus, Vienna Austria) microscope using the Volocity Software (Perkin Elmer).

The surface labelled with anti-GFAP, anti-Iba1, or Lectin was estimated as previously described [134, 135]. Briefly, z-stack pictures with a resolution of 1024 × 1024 pixels covering the whole 40 *μ*m slice were acquired using a LSM 700 confocal microscope (Carl Zeiss, Jena, Germany) with a 20x objective and Zeiss ZEN 2011 software. Stacks were merged using ImageJ Software 1.46r (National Institutes of Health, USA). The granular layer of dentate gyrus was outlined and converted to grey scale (Adobe Photoshop CS2, Adobe, San Jose USA) prior to the selection of a detection threshold. The percentage of pixels containing labelling within the dentate gyrus was quantified.

For statistical analysis, GraphPad Prism 5.0 (GraphPad Software Inc.) was used with two-tailed two-way ANOVA and Bonferroni post hoc test. Graphs show mean values with standard deviation as error bars. Significance was *P* < 0.05*, *P* < 0.01**, and *P* < 0.001***.
